# Long-term effect of persistent postpartum depression on children’s psychological problems in childhood

**DOI:** 10.1016/j.jad.2022.02.061

**Published:** 2022-02-24

**Authors:** Hanae Tainaka, Nagahide Takahashi, Tomoko Nishimura, Akemi Okumura, Taeko Harada, Toshiki Iwabuchi, Md Shafiur Rahman, Yoko Nomura, Kenji J. Tsuchiya

**Affiliations:** a Research Center for Child Mental Development, Hamamatsu University School of Medicine, Japan; b United Graduate School of Child Development, Osaka University, Kanazawa University, Hamamatsu University School of Medicine, Chiba University, and University of Fukui, Japan; c Department of Child and Adolescent Psychiatry, Nagoya University Graduate School of Medicine, Nagoya, Japan; d Queens College and Graduate Center, City University of New York, NY, United States

**Keywords:** Postpartum depression Internalizing problems, Externalizing problems, Cohort study

## Abstract

**Background:**

Maternal postpartum depression (PPD) is a well-established risk factor for psychological problems in children; however, little is known about the sustained impact of persistent PPD patterns and severity on these problems in children.

**Methods:**

Data were obtained from mothers (N = 714) and children (N = 768) from the Hamamatsu Birth Cohort for Mothers and Children. Maternal depression was measured using the Edinburgh Postpartum Depression Scale at 2, 4, 10 weeks and 10 months postpartum. Children’s internalizing and externalizing problems were assessed using the Strengths and Difficulties Questionnaire at 6 years and 8–9 years old. Mothers were divided into 4 groups based on the trajectory of their PPD persistence: “No PPD,” “Transient PPD,” “Worsening PPD” and “Persistent PPD.” Linear regression analysis was used to examine the association of PPD persistence and severity with children’s internalizing and externalizing problems.

**Results:**

“Persistent PPD” was significantly associated with children’s internalizing problems at 6 years old (Coefficient [95%CI] = 2.74 [1.30–4.19], P < .001), but no association was found at 8–9 years old. No associations were found between PPD severity and children’s internalizing and externalizing problems in either age category.

**Limitations:**

“Persistent PPD” and “Worsening PPD” groups had a relatively small sample size. The mothers’ depression statuses were not ascertained simultaneously with the children’s behavioral assessments. There was no information regarding the mothers’ treatment for PPD.

**Conclusion:**

PPD persistence negatively affected children’s internalizing problems but was not long-lasting. Future studies are needed to identify protective factors against PPD persistence in children’s psychological problems.

## 1. Introduction

Psychological and behavioral problems in childhood have negative impacts on children’s healthy development, academic functioning, and familial psychosocial function. Children’s psychological and behavioral problems have been classified into “internalizing problems” that include problems within oneself such as fears, bodily complaints, worrying, and social withdrawal and “externalizing problems”, which appear as behavioral problems such as disobedience, aggression, delinquency, temper tantrums, and overactivity (Achenbach, 1982). Preschool internalizing problems have been reported to lead to subsequent declines in cognitive and academic function (Bub et al., 2007) and a high risk of depression and other psychiatric disorders (Weeks et al., 2016). Externalizing problems in children and adolescents are a major risk factor in that they make subsequent psychosocial development and adaptation more difficult (Liu, 2004). Thus, identifying the risks concerning internalizing and externalizing problems is necessary for the healthy psychosocial development of children and helps inform effective early childhood interventions to mitigate or prevent subsequent mental health problems. One risk factor considered to be related to internalizing and externalizing problems is maternal postpartum depression (PPD) (Buckingham-Howes et al., 2017; Najman et al., 2000).

PPD is a common psychiatric disease observed in the postnatal period among mothers, with a reported prevalence of more than 20% (Howard et al., 2014). Most patients with PPD recover from the illness, but about one-third of them do not recover from PPD and develop chronic and severe depression (Murray et al., 2018). A recent study followed mothers until 40 months after delivery and showed that there were four trajectories of PPD involving: “High depression symptoms,” “Subclinical depression symptoms,” “Early postpartum depression symptoms,” and “Minimal depression symptoms” (Kingston et al., 2018). Another study, following mothers up to 38 months after delivery, identified four different trajectories of PPD: “High-persistent,” “Medium-decreasing,” “Low-increasing,” and “Low-stable” (Putnick et al., 2020). These studies support the need to investigate the different effects of PPD based on its persistence and severity when examining children’s suboptimal behavioral profiles.

Recent studies, including our own, suggest that PPD affects children’s neurodevelopment and subsequent mental health (Aoyagi et al., 2019; Murray et al., 2010; Murray et al., 2011; Sanger et al., 2015; Stein et al., 2014). Furthermore, some studies suggest that PPD affects children’s internalizing and externalizing problems (Kingston et al., 2018; Wang and Yan, 2019). These studies indicate that persistent and severe PPD has a particularly significant effect on children’s psychological and behavioral problems. For example, PPD has been found to have consistently adverse effects on children’s internalizing problems (Wang and Yan, 2019). However, concerning externalizing problems, study findings have been inconsistent as some studies have reported no effects of PPD on externalizing problems after controlling for confounding factors (Park et al., 2014; Vafai et al., 2016).

While it is important to consider PPD persistence, most previous studies have evaluated the effect of mothers’ PPD on children’s psychosocial problems at a single time point (Kingston et al., 2018; Vafai et al., 2016). Therefore, the sustained impact of persistence patterns of PPD on internalizing and externalizing problems has not been fully elucidated. In addition, it remains unclear whether PPD severity, especially among those for whom it is persistent, further affects children’s psychological and behavioral problems. Thus, in the present study, using data available from the Hamamatsu Birth Cohort for Mothers and Children (HBC Study), a longitudinal birth cohort study, we investigated whether the pattern of PPD (i.e., in terms of persistence and severity) was associated with children’s internalizing and externalizing problems.

## 2. Methods

### 2.1. Participants

The initial participants comprised mothers (n = 1138) and their children (n = 1258) who were born in Japan between December 2007 and June 2011. The recruitment procedures have been comprehensively described in a previous study (Takagai et al., 2016). The study procedures were approved by the Hamamatsu University School of Medicine and University Hospital Ethics Committee (research ID:17–037,19–145 and 20–233) and written informed consent was obtained from each mother to participate in the study. This study followed the STrengthening the Reporting of OBservational studies in Epidemiology (STROBE) reporting guidelines.

For the present study, we excluded mothers with data missing at one time or more in relation to four measurements conducted at 2, 4, 10 weeks, and 10 months after delivery using the Edinburgh Postpartum Depression Scale (EPDS), and 714 (62.7%) mothers and 768 (61.0%) children were included in the analysis.

### 2.2. Measurement

#### 2.2.1. Maternal depressive symptoms

Depressive symptoms were measured using the Japanese version of the EPDS (Cox et al., 1987) at 2, 4, 10 weeks and 10 months after delivery. The cut-off score for the EPDS was set as 9, which has been validated for clinical use in Japan (Ishikawa et al., 2011; Tamaki et al., 1997; Yamashita et al., 2000). In the American Psychiatric Association’s Diagnostic and Statistical Manual of Disorders, fifth edition (DSM-5) (American Psychiatric Association, 2013), perinatal depression is defined as depression that develops during pregnancy and within 4 weeks after delivery. However, previous studies have shown that maternal depressive symptoms develop not only within 4 weeks after delivery but also for up to 6 months after delivery (Bauman et al., 2020; Kettunen et al., 2014; Mori et al., 2011; Stewart and Vigod, 2016). Furthermore, the Word Health Organization and previous studies define the perinatal period as during pregnancy or within 12 months postpartum (Gavin et al., 2005; Putnam et al., 2017). Therefore, in this study, we defined PPD as depressive episodes that occur within 12 months postpartum and divided the participants into four groups, according to whether their EPDS scores were above the cut-off value at the time points of (1) 4 weeks or earlier, (2) 10 weeks, and (3) 10 months. The “No PPD” group consisted of children whose mothers with EPDS scores below the cut-off value at all 3 time points. The “Transient PPD” group consisted of children of mothers with EPDS scores above the cut-off value before 4 weeks or 10 weeks but below the cut-off value at 10 months. The “Worsening PPD” group consisted of children of mothers with EPDS scores below the cut-off value before 4 weeks or 10 weeks but above the cut-off value at 10 months. Finally, the “Persistent PPD” group consisted of children of mothers with EPDS scores above the cut-off value at all three time points. PPD severity was divided into “Moderate PPD” and “Severe PPD,” using EPDS scores at 10 months. “Moderate PPD” was defined as EPDS scores below 13 points, and “Severe PPD” was defined as EPDS scores above 13 points, as previously recommended (Cox et al., 1987).

#### 2.2.2. Children’s internalizing and externalizing problems

Children’s internalizing and externalizing problems were measured using the Japanese version of the Strengths and Difficulties Questionnaire (SDQ) (Goodman, 1997) via parental report when the children were 6 years and 8–9 years old. The SDQ consists of 25 items with 5 subscales: emotional symptoms, conduct problems, hyperactivity/inattention, peer problems, and prosocial behavior. Internalizing problems were calculated using the sum of scores for “Emotional Symptoms” and “Peer problems” and externalizing problems were calculated using the sum of scores for “Conduct Problems” and “Hyperactivity/Inattention” (Dickey and Blumberg, 2004; Goodman et al., 2010; Koskelainen et al., 2001).

#### 2.2.3. Covariates

The children’s sex, birth weight, maternal age at childbirth, maternal education level, household income, birth order, history of maternal affective disorder before delivery, and history of maternal affective disorder after delivery assessed at 40 months were included as covariates. Birth order was dichotomized into no previous live births (=0) and one or more previous live births (≥1). History of maternal affective disorder before delivery was assessed during pregnancy and maternal affective disorder after delivery was assessed at 40 months by trained clinicians using the DSM-fourth edition-text revision. If the mothers reported a history of major depressive disorder or bipolar disorder, they were categorized into an affective disorder group and the remaining mothers into a no affective disorder group. Model 1 was adjusted for child gender, birth weight, maternal age, maternal education level, household income, birth order, and history of maternal affective disorder before delivery. Model 2 was adjusted for all covariates in model 1 and the history of maternal affective disorder after delivery was assessed at 40 months.

### 2.3. Statistical analysis

All statistical analyses were conducted using Stata version 15. The Shapiro–Wilk test was used to examine whether scores of internalizing problems and externalizing problems were normally distributed. Although scores of internalizing problems were not normally distributed, visual inspection of the distribution was close to normal (see Supplementary Figs. 1 and 2), and the previous studies have indicated that the violation of the normality assumption should not cause major problems with large enough sample sizes (>30 or 40) (Ghasemi and Zahediasl, 2012). Therefore, we used the linear regression analysis in this study. The association of PPD persistence and severity with children’s internalizing and externalizing problems at 6 years and 8–9 years old was analyzed using linear regression analysis. The association of PPD severity with child internalizing and externalizing problems at 6 years and 8–9 years old was analyzed using linear regression analysis. Given that the participants in this study contained siblings born to the same mother, we used clustered robust standard error to calculate regression estimates. P-value significance was set at 0.004 (=0.05/12) using Bonferroni corrections for multiple testing (2 time points × 2 outcomes × 3 models). Power calculation was conducted using “Statistical power calculator” (α = 0.05, four levels, and effect size = 0.14 for small effect size) to determine the degree of confidence (https://www.statskingdom.com/33test_power_regression.html).

## 3. Results

### 3.1. Characteristics of the participants

A summary of the participants’ characteristics is provided in Table 1. The mean (standard deviation [SD]) maternal age at delivery was 32 (5.0) years, and the mean birth weight was 2952 (426.0) g. The characteristics of these participants did not differ from those identified in Japan’s national statistics (Takagai et al., 2016). Of the 768 children, 378 were boys (49.2%) and 390 were girls (50.8%).

Six hundred and forty-three children had mothers with “No PPD” (83.7%), 93 children (12.1%) had mothers with “Transient PPD,” 12 children (1.6%) had mothers with “Worsening PPD,” and 20 children (2.6%) had mothers with “persistent PPD.”

### 3.2. Association between PPD persistence and children’s internalizing and externalizing problems at 6 years old

In unadjusted models, internalizing problems were significantly associated with “Transient PPD” (Coefficient [95%CI] 1.04 [0.35–1.73], P = .003) and “Persistent PPD” (Coefficient [95%CI] =2.74 [1.30–4.19], P < .001) (Table 2). However, PPD persistence was not associated with externalizing problems. After controlling for confounders, a significant association was observed between internalizing problems and “Persistent PPD” only in Model 1 (Coefficient [95%CI] = 2.30 [0.85–3.76], P = .002) (Table 2). In Model 2, a marginally significant association between internalizing problems and “Persistent PPD” only was observed (Coefficient [95%CI] =2.09 [0.62–3.56], P = .005) (Table 2).

### 3.3. Association between PPD persistence and children’s internalizing and externalizing problems at 8–9 years old

At 8–9 years old, PPD persistence was not associated with internalizing and externalizing problems in either model (unadjusted, Model 1 and Model 2 in Table 3). A post-hoc power analysis showed that we had an adequate sample size to detect association with small effect size (statistical power = 0.81).

### 3.4. Association between severity of symptoms in mothers with persistent PPD and children’s internalizing and externalizing problems at 6 years and 8–9 years old

Considering that only “Persistent PPD” was associated with children’s internal problems, we focused on this group to examine the effect of PPD severity on children’s internalizing and externalizing problems. However, PPD severity was not found to be associated with internalizing and externalizing problems. (Table 4).

## 4. Discussion

In the present study, we showed that persistent PPD was associated with children’s internalizing problems at 6 years, even after controlling for confounding factors. However, this effect was not long-lasting, as there was no association found between persistent PPD and internalizing problems at 8–9 years old. Furthermore, there was no significant difference between PPD persistence and externalizing problems at both 6 years and 8–9 years old. We also found that the severity of symptoms in mothers with persistent PPD was not a risk factor for children’s internalizing and externalizing problems.

The present results are consistent with previous studies on the relationship between PPD and internalizing problems (Kingston et al., 2018; Wang and Yan, 2019) and expand the relevant literature by providing evidence indicating that the effect of PPD persistence is temporary. Recent studies suggest that PPD could continue for more than a few years after delivery (Putnick et al., 2020), and the “persistent PPD” group in our study may subsequently have become chronically depressed, with effects on the related children’s psychological development through inadequate attachment formation and nurturing attitudes in the longer term. Furthermore, previous studies have reported increased cortisol and/or reactivity to a stressor (Barry et al., 2015; Halligan, 2014) as well as dysfunction of the oxytocin system (Feldman, 2015) in children with mothers with PPD. Such biological changes derived from PPD in mothers could play a role in causing internalizing problems in children (Bender and Losel, 2021; Granger et al., 1998).

However, we did not find an association between PPD persistence and internalizing problems in the children at 8–9 years old. A previous study has shown that PPD in mothers at 8 months after delivery did not always have a negative effect in relation to children’s psychological problems when measured by the SDQ at 11 years of age (Savage-McGlynn et al., 2015). Therefore, our present results suggest that older children may have psychological plasticity in response to the effects of PPD, which is consistent with the work of Savage-McGlynn et al. In addition, there may be protective factors working against the negative effects of persistent PPD in relation to later psychological and behavioral problems in children. For example, Savage-McGlynn et al. reported that high nonverbal communication skills among children at 15 months and mothers’ positive perspective concerning their parenting at 18 months were associated with lower SDQ total difficulty scores at 11 years of age in children whose mothers had PPD at 8 months after delivery (Savage-McGlynn et al., 2015). In our previous study, we found that early-onset PPD affected the development of children’s nonverbal communication skills (Kawai et al., 2017). Therefore, it could be considered that PPD not only has direct effects on emerging psychological and behavioral problems in children but also that it has indirect effects in terms of the development of nonverbal communication skills. Another possible explanation for our results may be that because the number of participants differed between those aged 6 years and those aged 8–9 years old due to drop-out from the study, we may not have been able to clearly detect the effect of PPD on psychological and behavioral problems in children aged 8–9 years old. Further studies using a larger sample size and considering protective factors against the effect of PPD on psychological and behavioral problems are needed to clarify why the effect of PPD on children’s internalizing problems was limited to children aged up to 6 years old.

Although an association was found between persistent PPD and internalizing problems in children, PPD persistence was not associated with externalizing problems. These results were consistent with those of a previous study that showed that externalizing problems may not be influenced so much by PPD but rather by other factors, such as the mother’s current depression (Fihrer et al., 2009).

Contrary to our expectation, we did not find a significant association between the severity of PPD symptoms and internalizing and externalizing problems in children. Nets et al. showed that, regardless of the severity of the illness, PPD was associated with behavioral problems in children aged 3.5 years in a persistent PPD group. Our results are partially consistent with their study, although Nets did not categorize children’s behavioral problems into two categories as in this study. However, they showed that only persistent severe PPD was associated with the related children’s depression at the age of 18 years (Netsi et al., 2018). Future long-term follow-up studies of children of mothers with PPD are needed to clarify how the severity of maternal PPD influences the trajectory of children’s psychological functioning in our cohort.

### 4.1. Limitations

Our study has some limitations. First, the number of participants in this study was limited, especially in the “persistent PPD” and “worsening PPD” groups. Although a post-hoc power analysis showed that we had an adequate sample size which met the minimum requirement to attain 80% power, further studies with independent sample size is needed to replicate our findings. Second, this study did not have data indicating whether women received pharmacotherapy and psychological treatment, which may affect the course of the illness. Similarly, the study did not have data whether children received treatment or intervention, which may have influenced the results. Lastly, we did not assess the EPDS at 6 years and 8–9 years old after delivery, and so could not eliminate the direct effect of the mothers’ psychological status on the rating of children’s internalizing and externalizing problems as measured by the SDQ. In fact, the association between children’s internalizing problems at 6 years of age and persistent PPD was attenuated after controlling for mothers’ history of affective disorders after delivery assessed at 40 months.

## 5. Conclusions

In this study, we found that PPD persistence affected children’s internalizing problems; however, the effect was not long-lasting. Replication studies are needed to confirm whether the effect of PPD persistence on children’s psychological problems is limited in childhood.

## Supplementary Material

Supplementary material

## Figures and Tables

**Table 1:** Characteristics of participants.

Characteristics	No PPD	Transient PPD	Worsening PPD	Persistent PPD	Deference testing
				
	(N = 643) Mean (SD)	(N = 93) Mean (SD)	(N = 12) Mean (SD)	(N = 20) Mean (SD)	

Child sex (male)	319 (49.6%)	42 (45.2%)	5 (41.7%)	12 (60.0%)	P = .603
Birth order (first-born)	308 (47.9%)	62 (66.7%)	7 (58.3%)	11 (55.0%)	P = .008
Birth weight (g)	2958 (423)	2902 (389)	2870 (749)	3041 (435)	P = .43^a^
Maternal education level	14.0 (1.90)	14.1 (1.98)	15.1 (1.78)	13.8 (1.92)	P = .23^a^
At the time the household income (Million JPY)	6.31 (2.93)	5.75 (1.88)	5.95 (1.83)	5.26 (1.75)	P = .13^a^
Maternal age at child’s birth	32.0 (4.94)	31.9 (4.90)	32.9 (4.54)	32.4 (6.62)	P = .91^a^
History of maternal affective disorder before delivery (Yes)	45 (7.00%)	16 (17.2%)	4 (33.3%)	10 (50.0%)	P < .001
History of maternal affective disorder after delivery assessed at 40 months (Yes)	29 (4.50%)	15 (16.1%)	3 (25.0%)	8 (40.0%)	P < .001

Abbreviations: PPD, postpartum depression; SD, standard deviation; JPY. Japanese yen Unless stated, the P-values were obtained using Chi-square teste.

a P-values were obtained using one-way ANOVA.

**Table 2:** Association between PPD persistence and children’s internalizing and externalizing problems at 6 years old.

		Internalizing problems			Externalizing problems		
			
	Group	coefficient	P-value	95%CI	coefficient	P-value	95%CI

Unadjusted	No PPD (N = 573)	[Reference]		[Reference]	[Reference]		[Reference]
	Transient PPD (N = 81)	1.04	0.003*	0.35 1.73	0.48	0.227	−0.30 1.26
	Worsening PPD (N = 12)	0.27	0.722	−1.23 1.77	0.74	0.312	−0.70 2.18
	Persistent PPD (N = 17)	2.74	<0.001***	1.30 4.19	1.90	0.013	0.41 3.40
Model 1	No PPD (N = 573)	[Reference]		[Reference]	[Reference]		[Reference]
	Transient PPD (N = 81)	0.78	0.028	0.09 1.48	0.44	0.263	−0.33 1.21
	Worsening PPD (N = 12)	0.12	0.890	−1.53 1.76	0.97	0.182	−0.46 2.40
	Persistent PPD (N = 17)	2.30	0.002*	0.85 3.76	1.56	0.046	0.03 3.10
Model 2	No PPD (N = 573)	[Reference]		[Reference]	[Reference]		[Reference]
	Transient PPD (N = 81)	0.70	0.047	0.01 1.39	0.35	0.388	−0.44 1.13
	Worsening PPD (N = 12)	0.00	0.999	−1.71 1.72	0.84	0.266	−0.65 2.33
	Persistent PPD (N = 17)	2.09	0.005	0.62 3.56	1.32	0.113	−0.31 2.95

Model 1 included gender of children, birth weight, maternal age at child’s birth, maternal education level, at the time the household income, birth order, history of maternal affective disorder before delivery as covariates.

Model 2 included all covariates in model 1 and history of maternal affective disorder after delivery assessed at 40 months.

* P < .05

** P < .01

*** P < .001 after Bonferroni correction.

Abbreviations: PPD, postpartum depression; CI, confidence interval.

**Table 3:** Association between PPD persistent and child internalizing and externalizing problems at 8–9 years old.

		Internalizing problems			Externalizing problems		
			
	Group	coefficient	P-value	95%CI	coefficient	P-value	95%CI

Unadjusted	No PPD (N = 525)	[Reference]		[Reference]	[Reference]		[Reference]
	Transient PPD (N = 71)	0.90	0.028	0.10 1.71	0.31	0.491	−0.57 1.19
	Worsening PPD (N = 11)	1.20	0.051	−0.01 2.41	0.91	0.224	−0.56 2.37
	Persistent PPD (N = 16)	2.26	0.006	0.66 3.85	−0.49	0.528	−2.03 1.04
Model 1	No PPD (N = 525)	[Reference]		[Reference]	[Reference]		[Reference]
	Transient PPD (N = 71)	0.63	0.117	−0.16 1.43	0.29	0.504	−0.55 1.13
	Worsening PPD (N = 11)	0.83	0.201	−0.44 2.10	1.06	0.162	−0.43 2.54
	Persistent PPD (N = 16)	1.72	0.046	0.03 3.41	−0.96	0.263	−2.65 0.73
Model2	No PPD (N = 525)	[Reference]		[Reference]	[Reference]		[Reference]
	Transient PPD (N = 71)	0.56	0.157	−0.22 1.34	0.14	0.740	−0.69 0.98
	Worsening PPD (N = 11)	0.72	0.289	−0.61 2.04	0.83	0.282	−0.68 2.34
	Persistent PPD (N = 16)	1.51	0.089	−0.23 3.25	−1.39	0.159	−3.31 0.54

Model 1 included gender of children, birth weight, maternal age at child’s birth, maternal education level, at the time the household income, birth order, history of maternal affective disorder before delivery as covariates.

Model 2 included all covariates in model 1 and history of maternal affective disorder after delivery assessed at 40 months.

Abbreviations: PPD, postpartum depression; CI, confidence interval.

**Table 4:** Association between severity of symptoms in mothers with persistent PPD and children’s internalizing and externalizing problems at 6 years and 8–9 years old.

		Internalizing problems			Externalizing problems		
			
Child age	Group	coefficient	P-value	95%CI	coefficient	P-value	95%CI

6 years	Moderate PPD (N = 12)	[Reference]		[Reference]	[Reference]		[Reference]
	Severe PPD (N = 5)	2.73	0.031	0.28 5.18	−0.87	0.640	−4.72 2.99
8–9 years	Moderate PPD (N = 10)	[Reference]		[Reference]	[Reference]		[Reference]
	Severe PPD (N = 6)	3.13	0.059	−0.14 6.41	−1.13	0.469	−4.38 2.12

Abbreviations: PPD, postpartum depression; CI, confidence interval.
