# Supplementary material for: Long-term effect of persistent postpartum depression on children’s psychological problems in childhood
Source: J Affect Disord. Author manuscript; Available in PMC 2024 May 20. (PMC11105964; doi:10.1016/j.jad.2022.02.061)
Supplement: Supplementary material [file NIHMS1984925-supplement-Supplementary_material.docx]

**Supplementary Figure legends**

**Figure 1. Histogram of children’s internalizing and externalizing problems at 6 years old.**

X axis represents total scores of internalizing and externalizing problems of SDQ in children at 6 years old. Y axis represents the density.

Abbreviation: SDQ, Strengths and Difficulties Questionnaire

**Figure 2. Histogram of children’s internalizing and externalizing problems at 8-9 years old.**

X axis represents total scores of internalizing and externalizing problems of SDQ in children at 8-9 years old. Y axis represents the density.

Abbreviation: SDQ, Strengths and Difficulties Questionnaire

Figure 1. Histogram of children’s internalizing and externalizing problems at 6 years old.

Figure 2. Histogram of children’s internalizing and externalizing problems at 8-9 years old.
